# Molecular pathogenesis of myocardial remodeling and new potential therapeutic targets in chronic heart failure

**DOI:** 10.1186/1824-7288-38-41

**Published:** 2012-09-12

**Authors:** Giuseppe Distefano, Pietro Sciacca

**Affiliations:** 1 Department of Pediatrics, Pediatric Cardiology Service, University of Catania, Via S Sofia 78, Catania, 95123, Italy

**Keywords:** Chronic heart failure, Myocardial remodeling, Molecular therapeutic targets, Myocardial gene and regenerative therapy

## Abstract

It is well known that the natural history of chronic heart failure (CHF),regardless of age and aetiology,is characterized by progressive cardiac dysfunction refractory to conventional cardiokinetic, diuretic and peripheral vasodilator therapy. Several previous studies, both in animals and humans, showed that the key pathogenetic element of CHF negative clinical evolution is constituted by myocardial remodeling. This is a complex pathologic process of ultrastructural rearrangement of the heart induced by various neuro-humoral factors released by cardiac fibrocells in response to biomechanical stress connected to chronic haemodynamic overload. Typical features of myocardial remodeling are represented by cardiomyocytes hypertrophy and apoptosis, extracellular matrix alterations, mesenchymal fibrotic and phlogistic processes and by cardiac gene expression modifications with fetal genetic program reactivation. In the last years, increasing knowledge of subtle molecular and cellular mechanisms involved in myocardial remodeling has led to the discovery of some new potential therapeutic targets capable of inducing its regression. In this paper our attention is focused on the possible use of antiapoptotic and antifibrotic agents, and on the fascinating perspectives offered by the development of myocardial gene therapy and, in particular, by myocardial regenerative therapy.

## Introduction

Treatment of heart failure has been long based on three main drugs to augment contractile force and lighten the heart’s workload, i.e. cardiokinetics to correct ventricular contractile deficit, diuretics to eliminate hydric-saline retention and vasodilators to reduce increased systemic resistances caused by peripheral vasoconstriction. Hydric-saline retention and vasoconstriction result from the release of adrenergic amines, angiotensin and aldosterone in circulation due to the reflex activation of adrenergic and renin-angiotensin systems triggered off by the drop of intraaortic pressure determined by systolic output reduction [1,2]. However, this neuro-hormonal response aimed at maintaining peripheral circulatory pressure tone and ensuring vital organs perfusion further jeopardizes myocardial kinesis because it determines volume and pressure overload. Moreover, it explains the utility of diuretic and vasodilatative therapy to restore normal circulation.

## Physiopathology of chronic heart failure (CHF)

**Figure 1:**
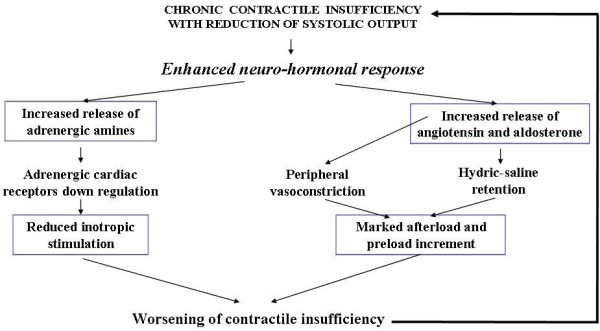
Vicious circle inpairing myocardial contractility in chronic heart failure.

In the chronic form of heart failure, whether idiopathic or secondary to various causes (inflammatory processes, ischemic syndromes, neuromuscular diseases, anthracycline induced toxicity, metabolic disorders, etc.) and generally linked to dilatative cardiomyopathies in childhood and adolescence, the therapeutic effectiveness of the above mentioned drugs tends to fade progressively over time. This may be because neuro-hormonal response linked to ventricular contractile deficiency can be overenhanced and thus severely damage heart function. In fact, while downregulation of adrenergic receptors resulting from the presence of excessive circulating amines inhibits adequate contractile response to adrenergic stimuli, increment of hydric-saline retention and intense peripheral vasoconstriction increase pre and after- load disproportionately. These effects result in more precarious heart performance giving rise to a vicious circle with progressive myocardial dysfunction that becomes more and more refractory to conventional treatment [3,4] (Figure 1). Molecular cardiology studies carried out in the last ten years have revealed some subtle pathogenetic mechanisms causing the progression of CHF and its resistance to traditional treatment [5]. It seems that in addition to the above-mentioned negative haemodynamic effects on pre and after-load, some pathologic modifications induced by adrenergic amines, angiotensin and aldosterone in the myocardial ultrastructure at cellular and extracellular level may be involved in the deterioration of cardiac functionality.

Furthermore, the same neuro-hormonal response can also be expressed to a greater degree inside the cardiac muscle and includes, together with catecholamines, angiotensin and aldosterone, other molecules such as endothelin, various proinflammatory cytokines (TNF-α, interleukin-β, interleukin-6) and some myocytic and vascular growth factors. These substances are released by cardiomyocytes after their mechano- receptors stimulation by biomechanical stress linked to chronic pressure and volume hemodynamic overload [6,7].

## Myocardial remodeling as leading cause of progressive cardiac dysfunction

**Figure 2:**
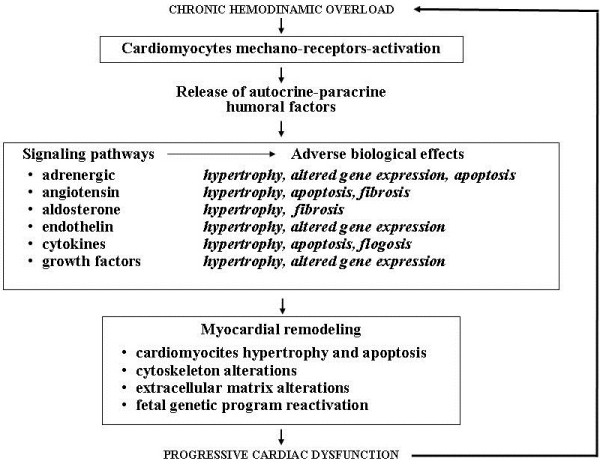
**Intramyocardial neuro-humoral response to biomechanic stress induced by chronic haemodinamic overload.** Release of humoral factors determinig myocardial remodeling and progression of cardiac dysfunction in chronic heart failure.

Numerous experimental studies have shown that the humoral factors secreted by mechanically stressed cardiomyocytes activate a vast network of intra and intercellular transduction signals. Acting in autocrine and paracrine fashion in the same cardiomyocytes and in the surrounding tissues, they are able to alter myocardial ultrastructure and determine cardiac fibrocells hypertrophy and/or apoptosis, mesenchymal fibrotic and inflammatory processes and induce modifications in cardiac gene expression [8-11]. This series of events is the basis of so called “myocardial remodeling” [12], i.e. a complex phenomenon of ultrastructural cardiac rearrangement that today, because of the sensible changes in cardiomyocytes viability, energetic metabolism and kinetic and electric properties and also in the cytoskeleton and extracellular matrix composition, is considered the key pathogenetic factor of CHF and of its natural history marked by inexorable, progressive cardiac dysfunction [13,14] (Figure 2).

One of the salient points in myocardial remodeling is altered cardiac gene expression, i.e. reactivation of fetal cardiac genetic program that can manifest with re-expression of genes that are hyperactive during the fetal period, including the gene of β-myosin (protein at low ATPasic activity poorly efficient in contractile function), some proapoptotic genes, and the gene of α subunit of Na,K dependent ATPase (an enzymatic variant having poor membrane stabilizing capacity). It may also involve inhibition of genes that are hyperactive in the adult heart, such as the genes of the sarcotubular ATPase (enzyme determining calcium reuptake from contractile filaments and hence essential for diastolic function), of β-adrenergic receptors (crucial for systolic activity), and of lipid β-oxidation (the main source of energy for the myocardium whose inhibition turns cardiac energetic metabolism into glycolysis that is poorly efficient for the heart) [15]. In recent studies on cardiac gene expression in various experimental models of cardiovascular diseases associated with heart failure, Kuwahara and Nakao [16] identified a series of “transcriptional pathways” involved in cardiac remodeling and connected to the reactivation of fetal cardiac genes implicated in the genesis of myocardial hypertrophy and severe cardiac rhythm disorders.

Therefore, remodeling related processes determine marked changes in myocardium phenotype that make it functionally more precarious. These changes include anomalies involving important molecules regulating systolic and diastolic function (as alfa- myosin and sarcotubular ATPase), cytoskeletal proteins and extracellular matrix composition. In the cytoskeleton, enhanced protein filament expression and increased microtubular network density can lead to sarcomere disorganization, while in extracellular matrix, fibroblastic hyperplasia and augmented collagen synthesis with production of rigid type 1 collagen fibrils can reduce ventricular compliance [17].

**Figure 3:**
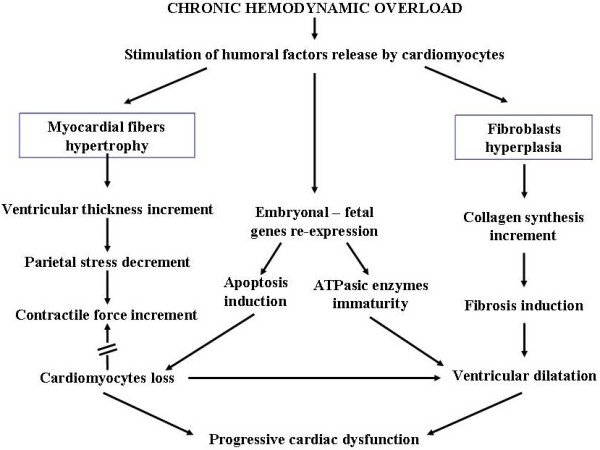
**Alterations induced by chronic biomechanic stress on cardiac muscle.** Note that beneficial effects of myocardial hypertrophy on cardiac performance are counteracted by induction of apoptotic and fibrotic processes.

**Figure 4:**
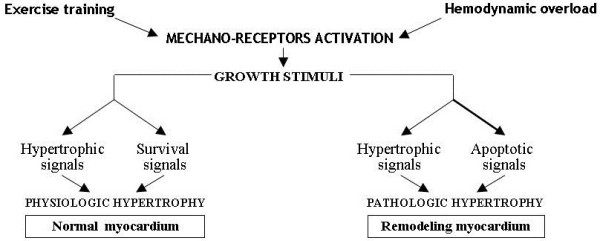
Different response to growth stimuli in physiologic and pathologic cardiac hypertrophy.

Instead of representing a useful compensatory event, in the long run also myocardial hypertrophy triggered off by chronic hemodynamic overload may damage the heart. Increased ventricular wall thickness initially reduces parietal stress (and hence oxygen requirement) and increases heart contractility. However, the compensatory effect is markedly curbed by subsequent re-expression of pro-apoptotic embryo-fetal genes and by fibroblastic hyperplasia with increased collagen synthesis, caused by the same biochemical mediators determining hypertrophy. Loss of contractile tissue due to cardiomyocytes apoptosis and mesenchymal fibrosis determined by increased collagen synthesis makes the heart dilate and stiffen, causing systolic and diastolic dysfunction [18] (Figure 3). Thus, the hypertrophy that occurs during cardiac remodeling differs from physiologically induced hypertrophy during exercise training where the growth signals of myocardial fibers are accompanied by biochemical signals favoring their trophism and survival. Diversely in remodeling, growth signals are associated with signals promoting cardiomyocytes apoptosis resulting in the loss of contractile elements that are replaced by fibrous tissue [19-21] (Figure 4). In addition, the two types of hypertrophy have different morphological expression. In physiological hypertrophy the cardiomyocytes grow evenly in length and width, whereas in pathological hypertrophy they become wider during pressure overload and longer during volume overload, and this can disrupt sarcomere alignment with inevitable negative repercussions on cardiac function [22]. Such acquisitions on cardiac hypertrophy heterogeneity have stimulated numerous studies aimed at better understanding of the subtle molecular mechanisms underlying the two types of hypertrophy, physiological and pathological, in order to draw useful therapeutic indications. A recent important review [23] indicates that the myocardium presents increased protein synthesis, cellular growth and increment of extracellular matrix in both pathological and physiological hypertrophy, whereas it differs greatly from a biochemical, metabolic and molecular viewpoint in the two types of hypertrophy. Pathological hypertrophy shows concomitant apoptotic, oxidative and inflammatory events, and glycolysis, that produces less energy than lipid beta-oxidation, prevails in energetic metabolism. On the contrary, in physiological hypertrophy lipid β-oxidation is accompanied by signaling pathways activation leading to the expression of biochemical mediators improving trophism and cardiomyocytes survival. Furthermore, it has been observed that most endocellular signals promoting physiological hypertrophy are linked to kinase AKT system activation, while those of pathological hypertrophy occur after G-protein system activation [23].

## Therapeutic strategies to reverse myocardial remodeling

The recent advances achieved in the knowledge of molecular pathogenetic mechanisms of CHF have paved the way for new therapeutic approaches aimed at reversing myocardial remodeling and combating apoptotic and fibrotic processes responsible, respectively, for cardiomyocytes loss and myocardial wall rigidity, and thus jeopardizing global heart function.

### Use of antagonists of neuro-humoral factors

This strategy represents the first approach to antiremodeling treatment and used antagonists of the various humoral factors(see above) released by cardiomyocytes and involved in myocardial remodeling : i.e. ACE-inhibitors and sartanics for angiotensin; spironolactone and natriuretic peptides for aldosterone; β- blockers for adrenergic amines; direct antagonists (bosentan) for endothelin; monoclonal antibodies and soluble fusion receptors for cytokines [12,24]. The most commonly used β-blocker is cardvedilol that reduces oxygen requirements by slowing heart rhythm and by decreasing afterload through peripheral vasodilatation. It also possesses an antioxidant action and recovers intrinsic cardiac inotropism by increasing the number of adrenergic receptors and determining overexpression of α-myosin, a protein at high ATPasic activity, in cardiomyocytes [25,26]. Anticytokines were used mainly against TNF-α which usually presents very high serum concentrations in patients with CHF. Nonetheless, it must be underlined that to date clinical experiences using all these drugs, alone or in combination, are rather fragmentary and the results do not always agree with the benefits found in animal studies. Moreover, it must be kept in mind that there are still no controlled prospective studies confirming long-term efficacy. The results obtained using endothelin antagonists are particularly disappointing [27], and those using anti-TNFα (both monoclonal antibodies and soluble receptors) are surprisingly modest even if this cytokine represents the most important biochemical marker of inflammatory processes in CHF [28-30]. On the other hand, pentoxfylline, an agent able to downregulate TNF synthesis and induce a wide immunomodulating action and vasodilatation, appears to be a potentially efficient anticytokine [31]. The scarce effectiveness of direct TNF antagonists is probably linked to the multiple biochemical mechanisms causing inflammation in heart failure and to the frequent combination of inflammatory and oxidative events. Hence, in the future better clinical results may be achieved using substances targeting multiple proinflammatory signals. In effect, preclinical trials in rats reported that some substances such as histone deacetylase inhibitors could represent an innovative and very promising class of therapeutic agents thanks to their broad anti-inflammatory spectrum associated to antiapoptotic and antifibrotic properties [32].

### Use of antiapoptotic and antifibrotic agents

The increasing knowledge of the biochemical signals activated during myocardial remodeling have recently focused scientists’ attention on agents that can directly reverse the damaging apoptotic and fibrotic processes that impair heart function [33,34].

**Figure 5:**
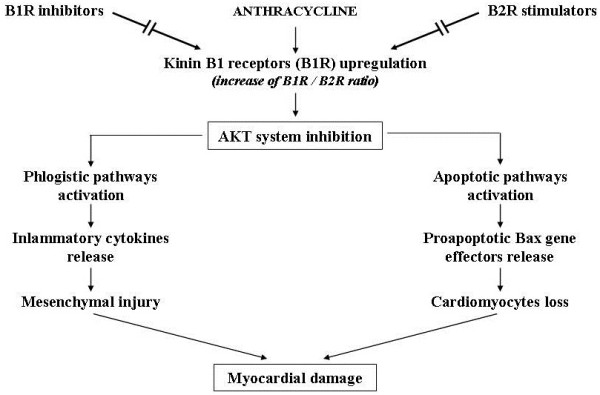
Potential therapeutic use of B1 and B2 kinin receptors (B1R, B2R) modulators to prevent anthracycline-induced myocardial damage.

**Figure 6:**
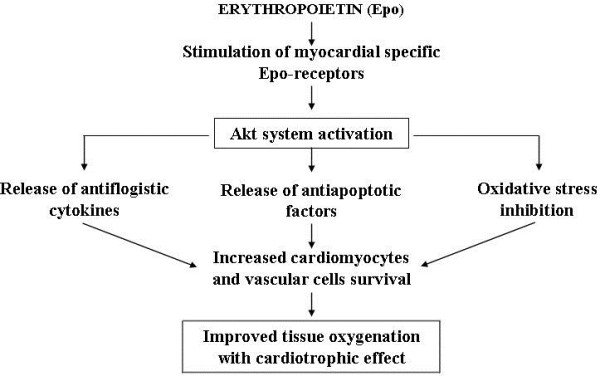
Possible mechanism by which erythropoietin is cardioprotective.

Concerning apoptosis, in various experimental animal models of CHF, apoptosis was hindered by substances that stimulate cardiomyocytes survival or inhibit death signals. Some potential therapeutic agents are cardiotrophin, a non inflammatory cytokine capable of activating GP-130-LIF protein and interrupting proapoptotic signals [22,35]; tetrandrine, an alkaloid isolated from a Chinese herb matrix that inhibits the ros-dependent ERC-1 signal that causes cardiac apoptosis and fibrosis [36]; and heme oxygenase-1 that frees carbon monoxide (CO) from the heme and seems capable of stabilizing mitochondrial membranes and inhibiting cytochrome C release that activates caspase enzymes inducing apoptosis. The beneficial effects of heme oxygenase-1 indicate the potential therapeutic use of CO-releasing molecules [37,38]. Other therapeutic advances against apoptosis use substances that activate the AKT biochemical system, which, as previously mentioned, protects cardiomyocytes and favors physiological hypertrophy, maintaining mitochondrial intactness [39]. Studies on anthracycline-induced chronic cardiomyopathy [40] postulated the use of molecules able to modulate cardiac bradykinin BR1 and BR2 receptors that respectively inhibit and stimulate the AKT system. It has been seen that one of the mechanisms by which anthracycline can damage the heart is linked to BR1 receptor upregulation (and increased BR1/BR2 ratio). Such upregulation suppresses the AKT system and favors inflammatory and apoptotic signals activation that can be blocked by BRI antagonists or BR2 stimulators [41] (Figure 5). Erythropoietin has also been used to treat anthracycline cardiomyopathy. It activates the AKT system and seems able to improve trophism and heart muscle oxygenation, and hence cardiomyocytes and vessels cells survival, by releasing anti-inflammatory, antiapoptotic and antioxidant factors [42] (Figure 6). Another molecule that seems to significantly activate the AKT system is IGF-1 and patients with CHF often present low serum IGF-1 levels correlated with the degree of systolic dysfunction [43]. However, to date there are no studies on the possible use of this substance.

As regards antifibrotic treatment, preclinical studies (in murine models) have frequently shown that fibrosis can be curbed by various substances, such as inhibitors of TGF-β, a growth hormone that stimulates myofibroblasts collagen synthesis [44] and torasemide. The latter is a loop diuretic capable of inhibiting procollagen type 1 carboxy- terminal proteinase and lysyl oxidase enzymes that play an important role in promoting rigid and insoluble type I collagen fibrils production and deposition [45,46]. Other useful substances are represented by the inhibitors of some specific micro ribonuleic acids whose overexpression is strictly associated with myocardial fibrosis [47].

## Future perspectives

Alongside these measures targeting apoptotic and fibrotic processes, the most noteworthy perspectives in the treatment of CHF are those connected to the development of myocardial gene therapy and myocardial regeneration therapy.

### Myocardial gene therapy

In the field of cardiovascular research the potential of gene therapy has been mostly explored in several models of inherited monogenic cardiac diseases. Recent insights concerning the molecular mechanisms and the identification of numerous genes and relative coded proteins involved in the pathogenesis of various acquired heart diseases have extended its application to heart failure.

Myocardial gene therapy consists of an intramyocardial transfer of specific genes for some molecular targets involved in myocardial remodeling through viral (mainly adenovirus) or non viral vectors (plasmids or oligonucleotids). Extensive preclinical studies have shown that this therapeutic approach is able to modulate calcium homeostasis in cardiac myocytes, manipulate adrenergic receptors related biochemical signals and increase cardiomyocytes resistance to apoptosis. The results of these studies foresee the potential of a “molecular ventricular assistance” in the failing heart [48].

Over the last few years, recent advances in myocardial gene therapy include improved vectors provided with greater trophism for myocardial cells and more efficient delivery methods, and have paved the way for translating experimental observations into therapeutic strategies in humans [49,50]. The first clinical trial targeting sarcoplasmic endoplasmin reticulum calcium ATPase (SERCA2a) was initiated after a satisfactory phase 1 study. This enzyme plays a crucial role in regulating calcium cycling (and hence modulating cardiac contractility) and is downregulated in CHF [51]. The SERCA2a gene was transferred via a recombinant adeno-associated virus vector in 39 patients with advanced CHF utilizing percutaneous intracoronary infusion as delivery method [52]. Recently, the results of this trial have conclusively shown positive biological outcome of the patients and have clearly demonstrated that SRCA2a is an important therapeutic target in CHF [53]. At present, clinical experimentation includes two other on-going trials targeting SERCA2a in the United Kingdom and in France and some targeting adenylyl-cyclase (AC6 isoform), an enzyme activated by beta-adrenergic receptors stimulation that plays an important role in cardiac inotropism and is downregulated in the failing heart [51].

Whether also the results of these studies confirm the clinical efficacy and safety of the treatment and other molecular targets (specially antiapoptotic signals) susceptible to gene manipulation will be successfully explored [54] in the near future, myocardial gene therapy will certainly represent a viable and important tool to improve cardiac performance in patients with CHF.

### Myocardial regenerative therapy

**Figure 7:**
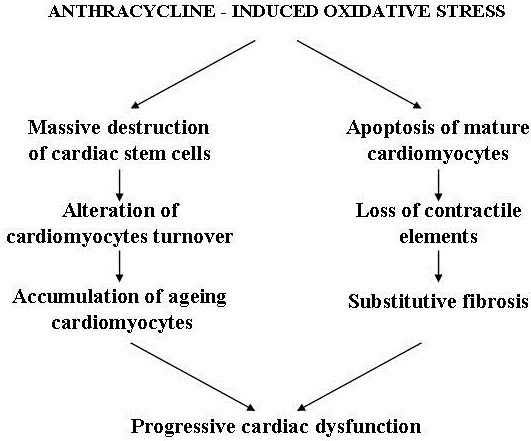
Effects of anthracycline on cardiac stem cells and on mature cardiomyocytes.

**Figure 8:**
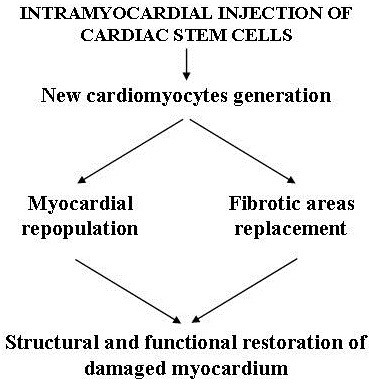
Effects of intramyocardial injection of cardiac stem cells in rats with anthracycline - induced cardiomyopathy.

Myocardial regenerative therapy has been adopted to replace the loss of cardiomyocytes and repair damaged myocardial tissue. This therapy is based on myocardial transplantation of stem cells derived from embryos, muscle, endothelium, bone marrow, etc. capable of differentiating into myocardial and endothelial cells and vascular myocytes under special conditions *in vitro.* The majority of these fascinating experimental researches have been carried out in acute and chronic ischemic cardiovascular pathologies and have mainly utilized bone marrow-derived mesenchymal stem cells that, together with embryonic-derived stem cells (rejected as they may determine teratomas), possess major cardiogenic potential [55]. However, results in clinical trials have not been very encouraging [56,57] and may be due to the adverse microenvironment in injured tissues of the failing heart (specially ischemia related). Indeed, the presence of inflammation, microvascular changes, altered oxygen tension and elevated levels of catabolites can impair not only the survival of stem cells inoculated into the myocardium, but also their differentiation into cardiomyocytes [58]. Nevertheless, recently great attention has been focused on myocardial regenerative therapy following reports that the adult heart of humans and other animal species is not a completely postmitotic organ, but possesses substantial regenerative potential. This is due to the presence of resident cardiac stem cells (RCSCs) capable of proliferating, differentiating into cardiomyocytes, endothelial and smooth muscle vascular cells, and migrating within the myocardium where they normally regulate cardiac cell homeostasis [59]. Studies in animals with ischemia-induced heart injuries revealed that intramyocardial injection of *in vitro* cultured RCSCs is able to promote myocardial and vascular tissue regeneration [60]. The utility of RCSCs therapy in CHF has been mainly taken into account for anthracycline-induced cardiomyopathy. Experimental *in vitro* and *in vivo* studies have revealed that because of their marked sensitivity to oxidative stress, these stem cells are particularly damaged by anthracycline, a well known producer of free radicals, and die more rapidly than mature cardiomyocytes. Furthermore, the ones that manage to survive malfunction in mature cardiac cells differentiation processes. Hence, in anthracycline-induced cardiomyopathy, massive RCSCs destruction damages physiological cardiomyocytes turnover and regeneration, leads to accumulation of ageing myocytes (mechanically less efficient) in the heart, and, at the same time, prevents the onset of repair processes of myocardial damage caused by apoptosis and fibrosis [61] (Figure 7). Intramyocardial injection of *in vitro* expanded RCSCs in rats with anthracycline-induced cardiomyopathy not only generated new cardiomyocytes and repopulated the heart with contractile elements, but also replaced fibrotic areas leading to structural and functional restoration of damaged myocardium [61] (Figure 8). It is clear that these results need to be confirmed by further studies corroborating the long term efficacy of regenerative therapy prior to their use in humans. If the results are positive, it is likely that intramyocardial injection of RCSCs, isolated from biopsy samples and expanded *in vitro* prior to anthracycline treatment, will represent a key therapeutic tool to prevent or treat severe heart dysfunction in patients with anthracycline-induced cardiomyopathy. In other forms of CHF where the RCSCs reserve pool is presumably not as severely impaired as in anthracyclinic cardiomyopathy, another emerging type of regenerative therapy could be intramyocardial delivery of trophic substances (various growth factors and non inflammatory cytokines) capable of stimulating cardiac stem cell proliferation and differentiation *in situ*[62].This therapeutic approach is less invasive as it does not require stem cells from endomyocardial biopsy for *in vitro* cultures and subsequent transplant. According to diverse experimental studies, such therapy exploits the fact that RCSC**s** possess specific receptors for these trophic substances and, once activated, not only proliferate and differentiate in myocardial and endothelial cells, but they also acquire the capacity to secrete trophic factors [63]. Acting in a autocrine and paracrine manner, these factors can further stimulate RCSCs growth and differentiation, and can also play a key beneficial role in myocardial remodeling. They achieve this by protecting cardiomyocytes against apoptosis, stimulating neoangiogenesis and inhibiting fibrosis, thus regulating extracellular matrix turnover [64,65]. The ability to produce trophic substances is not limited to RCSCs, but is shared by all stem cells, in particular mesenchymal stem cells (MSCs) [66]. It has been seen that such cells produce and secrete a large variety of cytokines, chemokines and growth factors [67] which all present elevated levels in their culture medium, regardless of the cell line they differentiate into [68]. Experimental studies on myocarditis-induced dilated cardiomyopathy in rats revealed the central role played by the paracrine effects of these humoral factors released by MSCs in reversing myocardial remodeling [69]. Other studies in animals with ischemic cardiac lesions demonstrated that intramyocardial delivery of conditioned medium (CM) from MSCs possesses a marked cytoprotective effect and favors cardiac repair. This protective effect on cardiomyocytes is greater when the medium is derived from genetically modified MSCs overexpressing the AKt-1 gene that is known to codify anti-apoptotic molecules synthesis [66]. These findings are of paramount importance in regenerative myocardial therapy as culture medium of stem cells derived from tissues that are easier to extract than heart tissue could be used as sources of trophic factors to stimulate RCSCs and activate repairing and cardioprotective processes. Experimental studies on perinatal hypoxic-ischemic brain damage in rats reported similar findings. In these investigations humoral factors present in culture medium of pluripotent stem cells derived from adipose tissue stroma were used to stimulate resident cerebral stem cells proliferation and differentiation for repairing cerebral damage [70,71]. In the heart, vascular endothelial growth factor and hepatocyte growth factor determine major RCSCs activation *in situ*, but recent studies in animals with acute and chronic cardiac ischemic lesions have also identified some synthetic molecules, for example hyaluronan mixed esters of butyric and retinoic acid, affording myocardial repair [65].

If all these extremely important experimental observations are confirmed by further studies, and if they can be reproduced in humans, in all likelihood intramyocardial delivery of a cocktail of natural and synthetic trophic substances capable of interacting positively in cardiac remodeling and myocardial regeneration can become the treatment of choice in CHF in the near future. Moreover, it may be the only therapy that can overcome the need for heart transplant that today represents the last chance for the survival of patients with this very severe disease. Finally, the combination of myocardial gene and regenerative therapy may achieve even better results.

## Competing interests

The authors declare that they have no competing interests.

## Authors’ contributions

GD conceived and designed the study. PS carried out the paragraphs “Physiopathology of chronic heart failure (CHF)”, “Therapeutic strategies to reverse myocardial remodeling “. Both authors read and approved the final manuscript.
